# Supplementary material for: γ-Cyclodextrin Metal-Organic Frameworks: Do Solvents Make a Difference?
Source: Molecules. 2023 Sep 29;28(19):6876. doi: 10.3390/molecules28196876 (PMC10574491; doi:10.3390/molecules28196876)
Supplement: Supplementary file 1 [file molecules-28-06876-s001.zip › molecules-2598084-supplementary.pdf]

Supporting Information

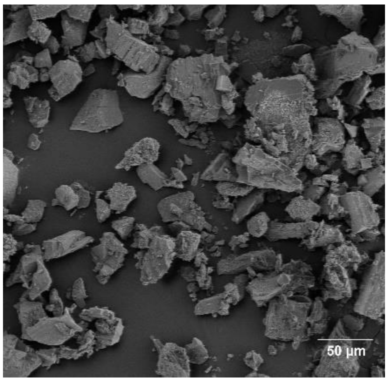

(**a**)

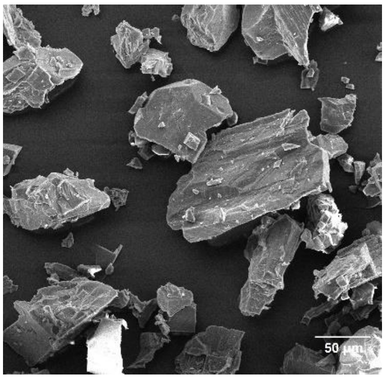

(**b**)

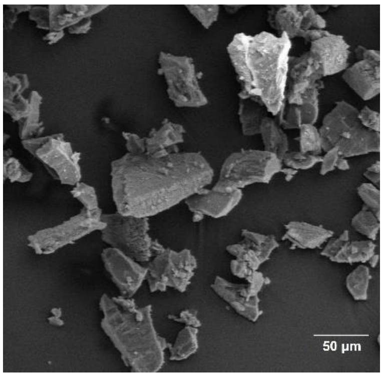

(c)

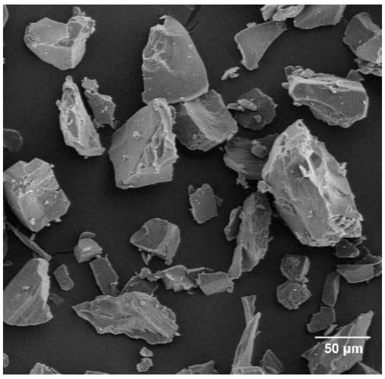

(d)

Figure S1. SEM images of: (**a**) curcumin-loaded γ-CD-MOFs (methanol-synthesised crystals, encapsulated via methanol), (**b**) curcumin-loaded γ-CD-MOFs (methanol-synthesised crystals, encapsulated via ethanol), (**c**) curcumin-loaded γ-CD-MOFs (ethanol-synthesised crystals, encapsulated via methanol), and (**d**) curcumin-loaded γ-CD-MOFs (ethanol-synthesised crystals, encapsulated via ethanol).

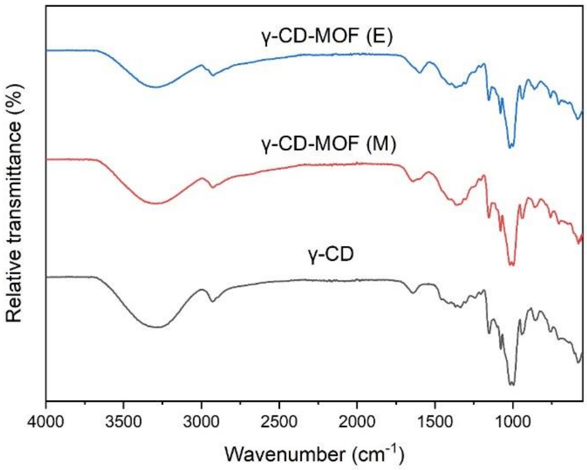

Figure S2. FT-IR patterns of γ-CD, γ-CD-MOFs (methanol-synthsised), and γ-CD-MOFs (ethanol synthesised).

The FT-IR spectra of γ-CD, methanol-synthesised γ-CD-MOFs, and ethanol-synthesised γ-CD-MOFs shown in Figure S2 have similar characteristic peaks. The strong, broad peak at 3000 – 3500 cm^{-1} indicated -OH stretching vibration; peak at approximately 1637 cm^{-1} accounted for a hydrate water vibration whereas 1360 cm^{-1} corresponded to -OH plane bending vibration. Thus, indicating that the change in synthesis solvent does not affect the intermolecular interaction in the γ-CD-MOFs significantly.

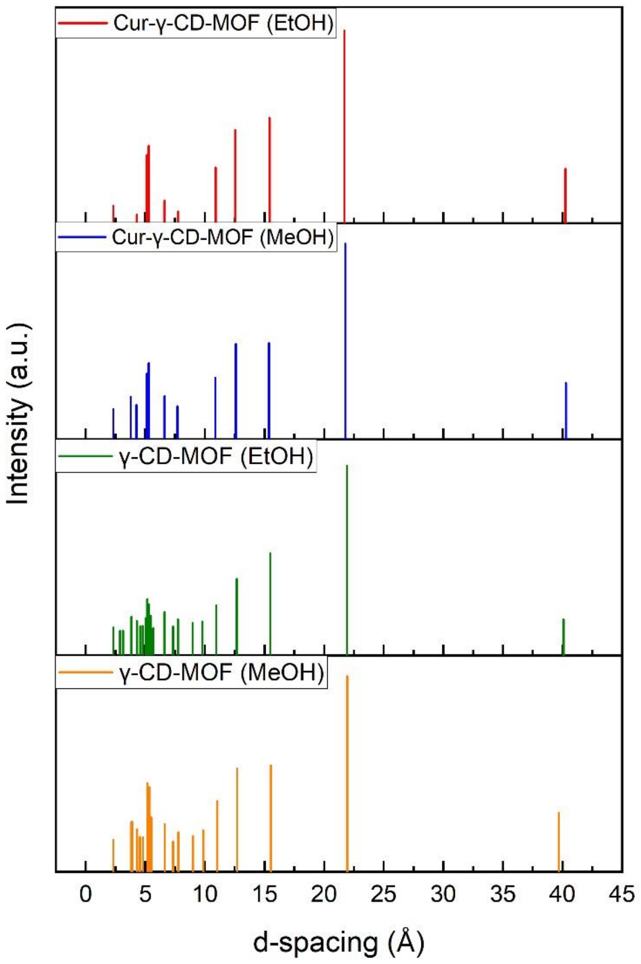

Figure S3. Diffraction peaks expressed in d-spacing of free γ-CD-MOFs and curcumin-loaded γ-CD-MOFs.
